# Evidence for Involvement of GIP and GLP-1 Receptors and the Gut-Gonadal Axis in Regulating Female Reproductive Function in Mice

**DOI:** 10.3390/biom12121736

**Published:** 2022-11-23

**Authors:** Dawood Khan, Opeolu O. Ojo, Orla RM Woodward, Jo Edward Lewis, Ananyaa Sridhar, Fiona M. Gribble, Frank Reimann, Peter R. Flatt, R. Charlotte Moffett

**Affiliations:** 1 Biomedical Sciences Research Institute, School of Biomedical Sciences, Ulster University, Coleraine BT52 1SA, Northern Ireland, UK; 2 Department of Biology, Chemistry & Forensic Science, School of Sciences, University of Wolverhampton, Wolverhampton WV1 1LY, UK; 3 Metabolic Research Laboratories, Wellcome Trust MRC Institute of Metabolic Science, Addenbrooke’s Hospital, University of Cambridge, Hills Road, Cambridge CB2 0QQ, UK

**Keywords:** Incretins, GLP-1R, GIPR, PCOS, fertility

## Abstract

Substantial evidence suggests crosstalk between reproductive and gut-axis but mechanisms linking metabolism and reproduction are still unclear. The present study evaluated the possible role of glucose-dependent-insulinotropic-polypeptide (GIP) and glucagon-like-peptide-1 (GLP-1) in reproductive function by examining receptor distribution and the effects of global GIPR and GLP-1R deletion on estrous cycling and reproductive outcomes in mice. GIPR and GLP-1R gene expression were readily detected by PCR in female reproductive tissues including pituitary, ovaries and uterine horn. Protein expression was confirmed with histological visualisation of incretin receptors using GIPR-Cre and GLP1R-Cre mice in which the incretin receptor expressing cells were fluorescently tagged. Functional studies revealed that female GIPR^−/−^ and GLP-1R^−/−^ null mice exhibited significantly (*p* < 0.05 and *p* < 0.01) deranged estrous cycling compared to wild-type controls, indicative of reduced fertility. Furthermore, only 50% and 16% of female GIPR^−/−^ and GLP-1R^−/−^ mice, respectively produced litters with wild-type males across three breeding cycles. Consistent with a physiological role of incretin receptors in pregnancy outcome, litter size was significantly (*p* < 0.001–*p* < 0.05) decreased in GIPR^−/−^ and GLP-1R^−/−^ mice. Treatment with oral metformin (300 mg/kg body-weight), an agent used clinically for treatment of PCOS, for a further two breeding periods showed no amelioration of pregnancy outcome except that litter size in the GIPR^−/−^ group was approximately 2 times greater in the second breeding cycle. These data highlight the significance of incretin receptors in modulation of female reproductive function which may provide future targets for pharmacological intervention in reproductive disorders.

## 1. Introduction

Two important aspects of animal physiology are metabolism and reproduction. These fundamental systems are interdependent with the hypothalamus acting as the control centre for reproductive function and energy intake [1,2]. Most hormones regulating feeding behaviour within the central nervous system (CNS) also contribute to the actions of gonadal hormones including estrogen [3,4]. Energy intake is a double-edged sword with both hypercaloric conditions such as obesity and energy deficiency leading to decreased fertility [5,6,7]. Furthermore, understanding hormonal control of energy homeostasis and fertility is essential for designing future treatments for reproductive disorders such as polycystic ovary syndrome (PCOS) which can affect up to one in ten women [7,8]. 

Glucagon-like peptide-1 (GLP-1) is a hormone produced by the preproglucagon gene and is predominantly expressed in the L-cells of the small intestine and, to some extent, in the CNS and pancreatic alpha-cells [9,10,11,12]. GLP-1R, a G protein-coupled receptor is expressed in various organs possibly including components of the reproductive system [13]. GLP-1 primarily regulates feeding, insulin secretion, glucose homeostasis, gastric motility and cardiovascular activity [12,14]. Recently, pharmacological stimulation of GLP-1R with liraglutide has been shown to significantly improve sexual symptoms, increase in total testosterone concentrations and recovery of hypothalamus-pituitary-testicular axis in humans [15]. In addition, GLP-1 also protects peripheral reproductive tissues against metabolic stresses during obesity, diabetes, and PCOS [13]. The sister incretin hormone, gastric inhibitory polypeptide (GIP), produced by intestinal K cells, is involved in insulin secretion, glucose homeostasis and both lipid and bone metabolism [11,16,17,18,19]. GIP also inhibits cortisol/corticosterone regulating enzyme 11β-hydroxysteroid dehydrogenase type 1 (11β-HSD1) [20]. Indeed, decreased hepatic 11β-HSD1 activity in obese women with PCOS highlights possible indirect effects of GIP in female fertility [21]. Similar to GLP-1R, the GIPR is expressed in the CNS, and possibly both ovaries and testes; suggesting its involvement in reproductive function [21]. Modulation of both GIPR and GLP-1R also significantly suppressed progesterone synthesis in the presence of follicle stimulating hormone (FSH) in rat granulosa cells [22]. 

Roux-en-Y bariatric surgery (RYGB), a gold standard treatment for weight loss, also corrects menstrual disturbances and ameliorates PCOS, leading to increased fertility [23,24,25]. Alterations of circulating gut peptides post RYGB and suggested expression of their receptors on hypothalamo-pituitary-gonadal reproductive axis [1,26,27,28] further indicate a functional correlation between energy homeostasis and reproductive function. However, the role of GIP, GLP-1 and their receptors in reproductive function and menstrual irregularities is scarcely studied and requires further investigation. Therefore, in the present study we evaluated the expression of GIPR and GLP-1R in the reproductive axis and their role in regulating estrous cycle, pregnancy outcomes and litter size in WT and transgenic mice with global knockout of either GLP-1R or GIPR. Finally, we investigated whether metformin treatment, an agent often prescribed in PCOS and which enhances GLP-1 action [12,29], may ameliorate pregnancy outcomes.

## 2. Materials and Methods

### 2.1. Experimental Animals

GIPR^−/−^ (GIPR KO) or GLP-1R^−/−^ (GLP-1R KO) female mice (14-weeks old), bred inhouse at Ulster University animal unit were used. These colonies were derived, respectively from animals kindly donated by Prof D Drucker (Toronto) and Prof B Thorens (Lausanne). The background and characteristics of these incretin receptor-deficient mice have been previously described [30,31]. Age-matched control WT mice from the same C57BL/6 genetic background were used as controls. Body weights, and non-fasted blood glucose and plasma insulin concentrations of control, GLP-1R KO and GIPR-KO female mice were not significantly different, consistent with earlier studies demonstrating plasticity of the incretin system [30,31,32]. Female 9–15 weeks old GLP1R-Cre × Rosa26-GCaMP3 and GIPR-Cre × Rosa26-GCaMP3 mice were used to provide evidence for incretin receptors on female reproductive tissues, thereby avoiding issues with the possible lack of specificity of antibodies for visualisation of these receptors by immunocytochemistry. The generation and characterisation of these mice have been detailed elsewhere [33,34]. Animals were caged individually and maintained on a 12/12 h light-dark cycle (lights on at 08h00, off at 20h00), in temperature-controlled room (T: 21.5 °C ± 1) with standard rodent diet and water ad libitum. All experiments were conducted in accordance with UK Animals Scientific Procedures Act 1986.

### 2.2. Assessment of Stages of Estrous Cycle

Assessment of estrous cycle stages was carried out as described previously [35]. Briefly, samples for wet smear were collected by flushing the vaginal fluid in mice using the tip of a plastic pipette filled with saline (~10 µL). The final flush containing vaginal fluid was placed on a glass slide and observed unstained under a light microscope (Olympus IX51, Olympus, Southend-on-Sea, UK) with ×10 objective lens without a cover slip. Classification of estrous cycle stages as proestrus, estrus, metestrus or diestrus was also carried out as documented [35]. Proestrus stage is characterized with predominant nucleated epithelial cells, which appears either in clusters or as individuals while estrus stage is distinctively characterized by cornified squamous epithelial cells, which occur in clusters. At metestrus, there is a mix of cell types with a predominance of leucocytes and a few nucleated epithelial and/or cornified squamous epithelial cells compared to diestrus stage which consists predominantly of leucocytes. Samples were collected and examined over a 20 days period and number of complete cycle of proestrus, estrus, metestrus and diestrus were computed for each mouse in the control, GIPR^−/−^ and GLP-1R^−/−^ groups. Number of cycles was evaluated by counting cycles where all four stages occurred during monitoring. The percentage of time spent in each stage was calculated by the number of days spent in each stage divided by the total number of days of monitoring.

### 2.3. Breeding Experiment

Reproductive function in GIPR KO, GLP-1R KO and WT mice was examined in groups of mice over 3-breeding periods of 21 days. Each female mouse was paired with age matched C57BL/6 male mouse throughout the breeding periods. Number of mouse-pairs producing pups, litters and litter-size for each breeding period were recorded the next day after birth. At the end of the first three breeding periods, all animals (male and female) were treated with metformin (approximately 300 mg/kg body weight), dissolved in the drinking water (1.109 g/L) for another two breeding periods to assess the effect of metformin treatment on the reproduction patterns of animals [36].

### 2.4. Gene and Protein Expression Studies

Tissues from GLP1R-Cre and GIPR-Cre x Rosa26-GCaMP3 mice were fixed for ~48 h in paraformaldehyde solution (4% *w/v* in phosphate-buffered saline) to preserve cellular architecture by cross-linking proteins. The tissues were then processed in an automated tissue processor which involved dehydrating tissues in 70% to 100% ethanol, followed by xylene immersion to remove wax before paraffin embedding. The tissues were then sliced into 5 μm slices and placed on poly-l-lysine coated slides. To assess staining GFP sections were dewaxed in histoclear for 30 min before being rehydrated with decreasing concentrations of ethanol. The sections were blocked with 2.5% bovine serum albumin (BSA) and then incubated with a primary antibody (GFP, 1:400, Abcam, ab5450, Cambridge, UK) overnight. On day 2, the sections were rinsed in phosphate-buffered saline (PBS) twice and incubated with secondary antibody (IgG Donkey Goat, Alexa Fluor^®^ 488, 1:400, Invitrogen, Hempstead, UK) for 1 h at 37 °C. After two more PBS washes, they were incubated with DAPI for 15 min at 37 °C followed by a last set of washes with PBS. Finally, the sections were mounted using antifade and coverslips. Stained sections were viewed at 10–20 × magnification using an Olympus system microscope, model BX51 and photographed using a DP70 digital camera system (Diagnostic Instruments Inc, Sterling Heights, MI, USA).

For gene expression studies, mRNA was extracted from pituitary, ovaries, and uterine horn excised from normal female C57BL/6 mice using RNeasy mini kit (Qiagen, Manchester, UK) according to the manufacturer’s instructions. mRNA (1–5 µg) was converted to cDNA using Superscript II reverse transcriptase RNase H kit (Invitrogen, Hempstead, UK). Quantifast SYBR green PCR kit (Qiagen, Manchester, UK) was used for real time reverse transcription PCR, with reaction mix containing 12.5 µL PCR master mix, 1 µL primers (forward and reverse, Invitrogen, Hempstead, UK), 1 µL cDNA and 9.5 µL RNase free water. Amplification conditions were initial denaturation at 95 °C for 5 min, final denaturation at 95 °C for 30 s, annealing at 58 °C for 30 s, extension at 72 °C for 30 s, with melting curve analysis at temperature range of 60 °C−90 °C. Each PCR experiment included a negative control and a positive control (Actb). MiniOpticon two-colour real time PCR detection system was used for data acquisition. Results were analysed using ΔΔCt method, with mRNA expression normalised to Actb. MIN6 cells were used as positive control as this cell line is well known to exhibit receptors for both GLP-1 and GIP. Sequences of primers used are contained in Table 1.

### 2.5. Statistics

Statistical analysis was performed using GraphPad PRISM (La Jolla, CA, USA; version 5). Data are presented as mean  ±  SEM for a given number of observations (n) as indicated in the Figure legends. Differences between groups were compared using one-way ANOVA or unpaired 2-tailed Student’s t test as appropriate. Statistical significance was accepted at *p* < 0.05.

## 3. Results

### 3.1. Expression of Genes for GIPR and GLP-1R in Female Reproductive Tissues of C57BL/6 Mice

**Figure 1:**
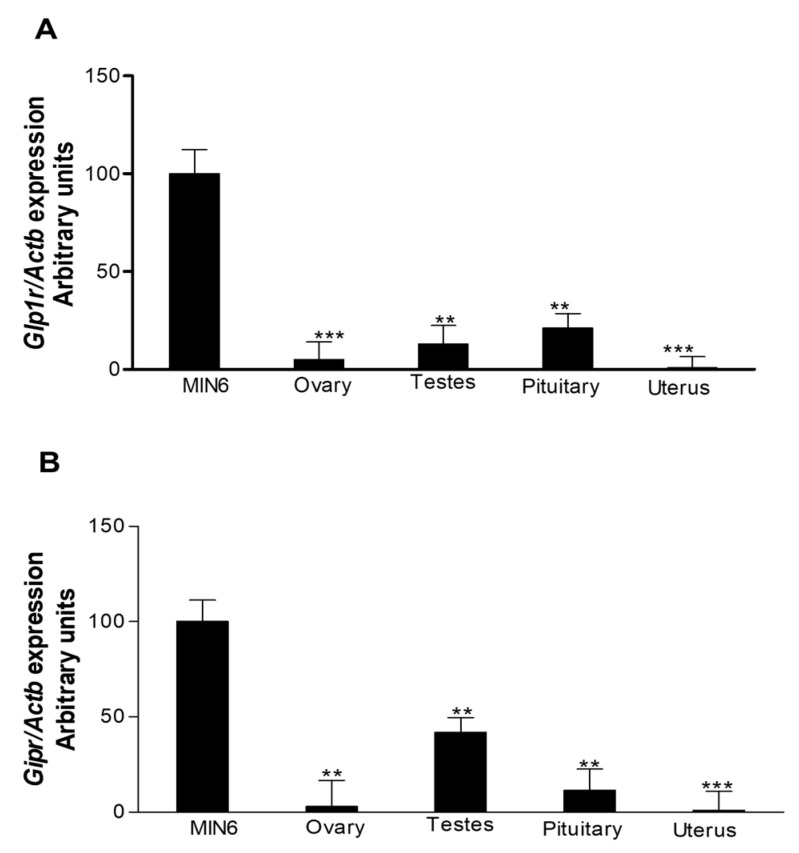
GLP-1 and GIP receptor gene expression in pituitary, ovary and uterine horn of female C57BL/6 mice. Relative mRNA expression (in %) in the pituitary, ovary and uterine horn of C57BL/6 mice. (**A**) GLP-1R and (**B**) GIPR mRNA expression. Values are mean ± SEM with *n* = 4. ** *p* < 0.01, *** *p* < 0.001 compared to insulin-secreting MIN6 cells.

Genes for GLP-1 and GIP receptors were expressed in the pituitary, ovary and uterine endometrium (Figure 1). Levels were significantly (*p* < 0.001 to *p* < 0.01) lower than expression in insulin-secreting mouse insulinoma MIN6 cells which were used as positive control (Figure 1).

### 3.2. Histological Localization of Incretin Receptors in Reproductive Tissues of Female GIPR-Cre and GLP1R-Cre Mice

**Figure 2:**
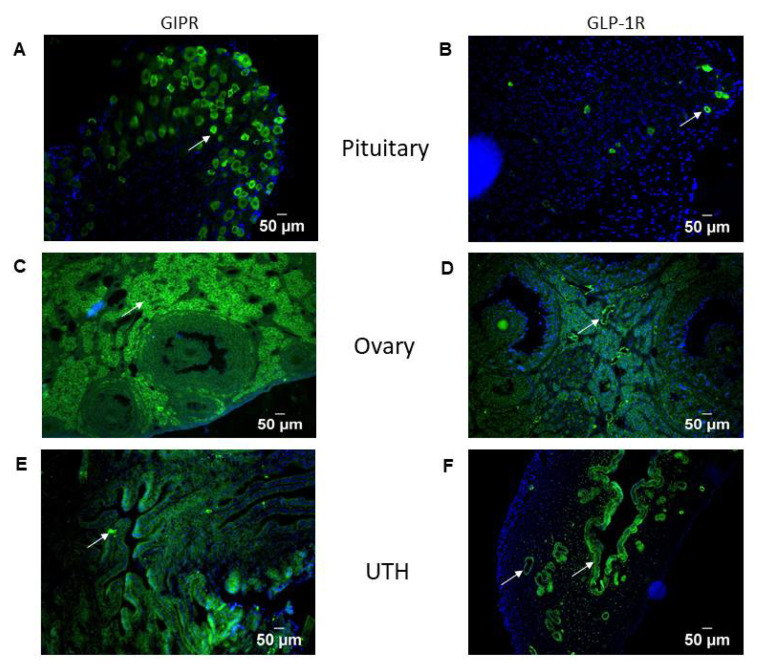
GLP-1 and GIP receptor protein expression in pituitary, ovary and uterine horn of female GIPR-Cre and GLP1R-Cre mice. (**A**–**F**) Representative histological images of pituitary, ovary and uterine horn (UTH) showing GIPR/GLP-1R (green, indicated by arrows) staining with DAPI (blue) demonstrating nuclei. Representative images shown with scale bars.

Histological evaluation in female GIPR-Cre and GLP1R-Cre mice revealed expression of both types of incretin receptors in pituitary, ovary and uterine horn (Figure 2A–F). Figure 2A,B shows positive staining for GIP and GLP-1 receptors in anterior pituitary cells. GIP and GLP-1 receptor immunostaining was observed in various parts of ovarian tissue except corpus luteum (Figure 2C,D). Figure 2E shows GIPR immunoreactivity predominantly in the luminal epithelium. Positive staining for GLP-1R is observed in uterine glands, luminal epithelium, intersection zone and stroma as shown in Figure 2F.

### 3.3. Stages of Estrous Cycle in GIPR^−/−^ and GLP-1R^−/−^ Mice

**Figure 3:**
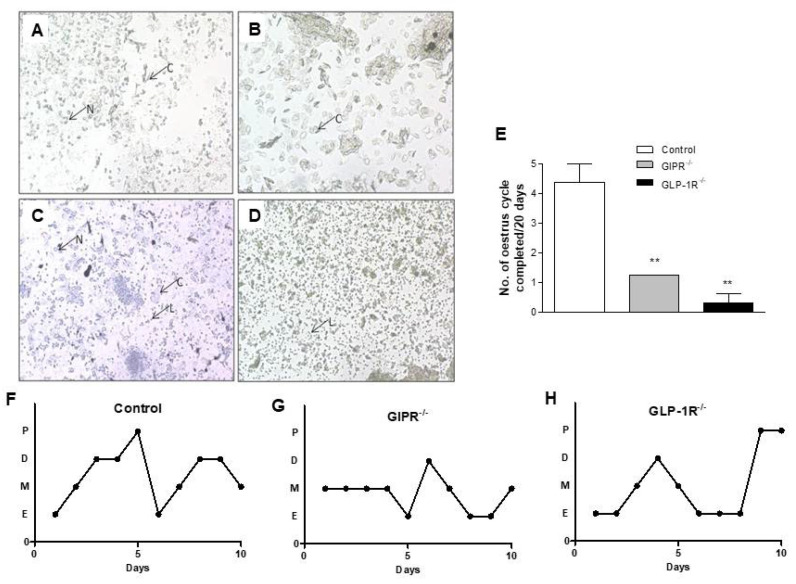
Menstrual stages in normal C57BL/6 mice and effects of GIP- and GLP-1-receptor knockout on estrous cycle. Representative photomicrographs of unstained vaginal smear from control C57BL/6 mice at proestrus ((**A**), consisting of mostly nucleated epithelial cells), estrus ((**B**), with predominantly anucleated cornified cells), metestrus ((**C**), consisting of leukocytes, cornified and nucleated epithelial cells) and diestrus ((**D**), containing mostly leucocytes). N = Nucleated epithelial cells, C = Cornified epithelial cells and L = Leucocytes. (**E**) Number of estrous cycles completed in 20 consecutive days. Graphic representation of different stages of first ten days of estrous cycle monitoring of individual (**F**) control, (**G**) GIPR^−/−^ and (**H**) GLP-1R^−/−^ mice. Values are mean ± SEM with *n* = 4. ** *p* < 0.01 compared to control.

Different stages of estrous cycle were recorded in vaginal smears for 20 consecutive days in control, GIPR^−/−^ and GLP-1R^−/−^ mice. Representative photomicrographs of each stage of the estrous cycle are shown (Figure 3A–D). The duration of each cycle was considerably longer in GIPR^−/−^ and GLP-1R^−/−^ mice compared with control animals. Female GIPR^−/−^ and GLP-1R^−/−^ mice exhibited significantly (*p* < 0.01) deranged estrous cycle compared to WT controls, indicative of reduced fertility (Figure 3E). Only 25% of GLP-1R^−/−^ mice completed a full estrous cycle during the 20 days period. Each GIPR^−/−^ female mouse completed just 1 cycle over this period. GIPR^−/−^ mice spent 10% excess time in metestrus stage while GLP-1R^−/−^ mice had 12% increased time in estrus stage compared to control mice suggesting a trend towards persistent estrus or persistent vaginal cornification (PVC). GIPR^−/−^ mice exhibited 11% reduction in percentage time in proestrus stage whereas GLP-1R^−/−^ mice showed 10% reduction in diestrus stage compared to the control group. Graphic representation of different stages of estrous cycle for first ten days of monitoring in a representative animal from control, GIPR^−/−^ and GLP1-R^−/−^ groups is shown (Figure 3F–H). These observations indicate that loss of functional receptors for GIP or GLP-1 results in significant derangements in estrous cycling, possibly affecting fertility.

### 3.4. Reproduction Outcomes in Control, GIPR^−/−^ and GLP-1R^−/−^ Mice

**Figure 4:**
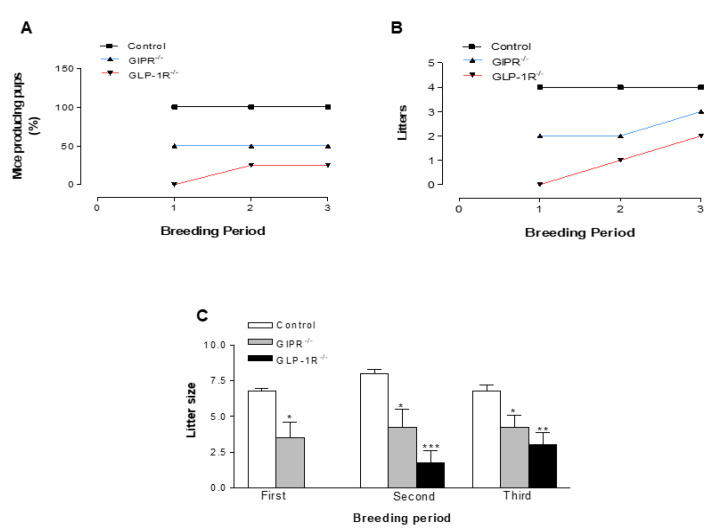
Percentage of animals producing pups, litters and litter size in WT, GIPR^−/−^ and GLP-1R^−/−^ mice. Female (WT, GIPR^−/−^ or GLP-1R^−/−^) mice (*n* = 4) were bred with age-matched normal male C57BL/6J mice over three breeding periods of 20 days each. (**A**) Percentage of mice producing pups, (**B**) litters produced and (**C**) average litter size in female WT, GIPR^−/−^ and GLP-1R^−/−^ mice. Litter size corresponds to number of pups in each litter whilst litter refers to number of litters produced. Values are mean ± SEM with *n* = 4. * *p* < 0.05, ** *p* < 0.01, *** *p* < 0.001 compared to control.

**Figure 5:**
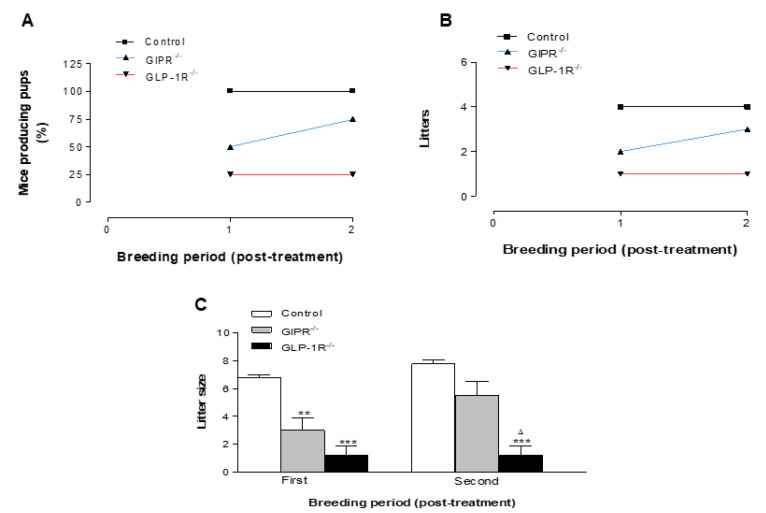
Percentage of animals producing pups, litters and litter size in WT, GIPR^−/−^ and GLP-1R^−/−^ mice treated with metformin. Female (normal, GIPR^−/−^ or GLP-1R^−/−^) mice were bred with age-matched normal male C57BL/6 mice over two breeding periods of 20 days each. All animals were treated with metformin (300 mg/kg body weight) dissolved in drinking water throughout the period of the experiment. (**A**) Percentage of mice producing pups, (**B**) litters produced and (**C**) average litter size in female WT, GIPR^−/−^ and GLP-1R^−/−^ mice post metformin treatment. Litter size corresponds to number of pups in each litter whilst litter refers to number of litters produced. Values are mean ± SEM with *n* = 4. ** *p* < 0.01, *** *p* <0.001 compared to control. Δ *p* < 0.05 compared to GIPR^−/−^.

**Figure 6:**
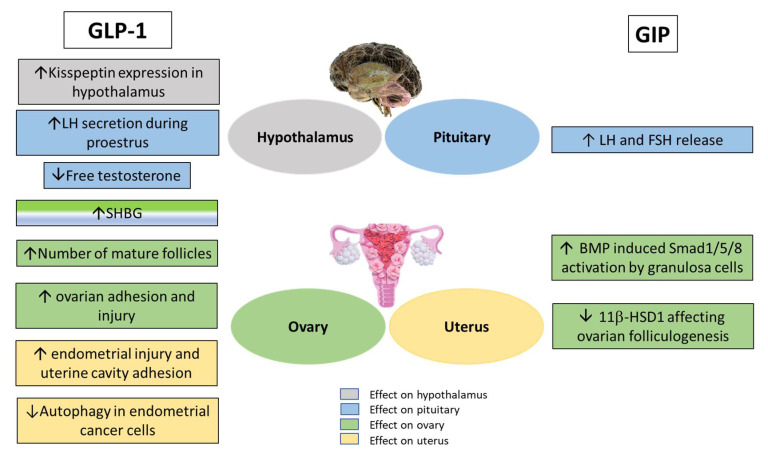
Schematic model showing effects of GLP-1 and GIP receptor modulation on female brain-reproductive function. Arrow up signifies increase/improves; arrow down signifies decrease. Abbreviations used: BMP-bone morphogenetic protein; Smad1/5/8-transcription factors; 11β-HSD-11β-Hydroxysteroid dehydrogenase.

Reproductive outcome showed an average reduction of 50% and 83% in the number of mice producing pups at all breeding periods in GIPR^−/−^ and GLP-1R^−/−^ mice, respectively, compared to WT control (Figure 4A). The control group produced an average of between 4–5 pups litters during the 5 breeding periods (Figure 4B and Figure 5B). This was significantly (*p* < 0.001) greater compared with average of 2–3 and 1 litters observed for the GIPR^−/−^ and GLP-1R^−/−^ groups, respectively (Figure 4B and Figure 5B). Moreover, the average litter size for each pregnancy produced by GIPR^−/−^ and GLP-1R^−/−^ mice was significantly (*p* < 0.001 to *p* < 0.05) lower compared to WT control (Figure 4C and Figure 5C). Treatment with oral metformin (300 mg/kg body weight) produced no significant change in reproduction outcome (Figure 5A) except that litter size in the GIPR^−/−^ group on metformin treatment was 1.8 times greater in the second breeding cycle (Figure 5C). These observations support a hitherto unrecognized role of GIP and GLP-1 play a role in female fertility. Figure 6 shows schematic diagram of effects of incretin hormones GLP-1 and GIP receptor modulation on brain-reproductive axis.

## 4. Discussion

The most widely accepted biological functions of the incretin hormones GLP-1 and GIP include increasing satiety, inhibiting gastric emptying, stimulating glucose-dependent insulin secretion, suppressing glucagon secretion and enhancing islet function, thereby playing an important role in maintaining body weight and blood glucose homeostasis [11,12,16,27]. However, the expression of these peptide receptors in pituitary and other elements of the reproductive axis, as shown definitely by this study and suggested previously [8,13,22,37], indicates a possible direct or indirect role of these gut peptides in regulating reproductive function.

Consistent with previous studies [34,38], GIPR and GLP-1R gene expression was detected in pituitary, ovary and uterine horn of WT C57BL/6 mice. However, more important than simple identification of the genes, our histological analysis using GLP-1R- and GIPR-Cre mouse models [33,34], definitively identified cells expressing receptors for GLP-1 and GIP. Similar to observations of GIPR in neuronal and non-neuronal cell types [34], we reported notable GIP and GLP-1 receptors in the pituitary gland. Interestingly, a study has also reported increased GLP-1R mRNA levels in rodent hypothalamus and ovaries as well as low expression levels in pituitary [34]. Indeed, we found that both GIP and GLP-1 receptors were present in the ovaries and uterine horn, suggesting their involvement in important aspects of female reproductive function. Previous studies have also demonstrated GLP-1Rs in mouse ovarian granulosa cells [39].

GLP-1R-Cre mice have been used as a functional tool to map the distribution of GLP-1-R [34] and GIP-R [40] throughout the murine brain. Since the hypothalamus is an important regulator of pituitary, adrenal and ovarian function, further studies are required to assess whether incretin hormone receptors can affect reproductive function at the level of hypothalamus as well as directly at the pituitary, ovary and uterine endometrium. For example, possible effects at the latter site could influence the implantation, development and the nurture of fertilised ovum. Overall, these combined observations suggest that both GLP-1 and GIP modulate gonadotropic axis at the level of the pituitary and ovary as well as the affecting the normal physiology of the uterine endometrium.

Consistent with this view, genetic deletion of both GLP-1R and GIPR significantly disturbed estrous cycles in mice with GLP-1R deletion causing greater disturbances compared to WT controls. This is presumably driven by disturbances of FSH, LH, estrogen, progesterone and/or other hormones involved in regulation of ovarian function and changes in uterine and vaginal morphology. Future detailed studies evaluating circulating reproductive hormones are required to fully elucidate the involvement of these factors in the disturbed estrous cycling and reproductive performance in these mice.

GLP-1R deleted mice spent greater proportion of the cycle in estrus stage, this persistent estrus or prolonged vaginal cornification (PVC) is associated with anovulation, reproductive senescence, and impaired fertility [41,42]. GLP-1R deleted mice also showed significant reduction in the number of mice producing pups. MacLusky et al. reported no obvious reproductive deficit in GLP-1R^−/−^ mice but used groups of 8 transgenic mice compared with 22 for controls [26]. Studies also suggest the possibility of prolonged periods of estrus linked to disrupted hypothalamic activity [43,44,45]. GIPR deletion in mice also revealed significant disturbance in cycle length with increased portion of estrous cycle spent in metestrus and diestrus stages. The expression of incretin hormones encoding genes and their receptor in several regions of brain is indicative of a plausible mechanism for incretin receptor-induced estrous cycle regulation [12,16,38].

Further studies are required to explore these aspects but the possibility is supported by previous reports of significantly improved menstrual frequency and ovulation rate in women with PCOS treated with GLP-1R agonists and DPP-4 inhibitors [46,47,48,49,50,51,52]. Our data call for further detailed studies of the mechanisms through which incretin hormones affect female reproductive function, including observations using tissue specific ablation of incretin receptor genes at various sites of the hypothalamic–pituitary–gonadal axis. In agreement with our observations, MacLusky et al. (2000) reported that global genetic deletion of GLP-1R decreased number of ovarian follicles and delayed onset of puberty in mice [26]. Furthermore, intraventricular injection of GIP has been shown to decrease serum FSH levels [1] in rats. However, the cellular mechanisms through which GIP, GLP-1 and their receptors contribute to normal reproductive function and irregular menstruation are largely unknown.

Based on the actions and use of metformin in the clinical treatment of PCOS [53], we evaluated the potential ability of metformin to improve fertility in mice. In addition, previous studies show that metformin treatment enhances the expression of the genes encoding the receptors for both GLP-1 and GIP highlighting possible incretin-sensitizing effects of metformin [54,55]. A previous study also showed increased circulating GLP-1 in obese humans treated with metformin [56]. As such, daily administration of metformin resulted in marginally improved litter outcome and fertility in GIPR^−/−^ mice in the second breeding cycle. However, metformin had no effect on GLP-1R^−/−^ mice. This result accords with the view that in addition to stimulation of AMPK [12,57], the biguanide mildly activates the GLP-1 system either by stimulating its secretion [12], inhibiting its degradation by DPPIV or both [58]. Metformin in non-diabetic individuals increased GLP-1 independent of changes in weight and glycaemia, similarly, the mild reproductive effects observed with metformin in our study are unlikely to reflect improvements in metabolic control as the untreated KO mice exhibited normal glucose and insulin levels [59]. In agreement with role of GLP-1 in female fertility, treatment with GLP-1 and exendin-4 also increased the number of implanted foetuses and pups born in female rats [34]. Interestingly, a previous study published in our laboratory suggested a key role of GLP-1R and GIPR deletion in islet-derived hormonal adaptation during pregnancy [32]. Other reports have also indicated the possible involvement of additional metabolic peptides including PYY, ghrelin, leptin, and adiponectin in the regulation of reproductive function [7,8,60,61]. Further investigation utilising stable analogues of these peptides and incretin hormones is required to fully understand the involvement of gut-reproductive axis in female fertility.

## 5. Conclusions

Taken together, our observations along with previously published literature [1,7,8,13,61], provide evidence that both GLP-1R and GIPR are present in tissues of the gut-gonadal axis and play an important role in regulating energy-linked reproductive function and preserving female fertility. Effects of these key receptors may be manifested by the interactions shown in Figure 6. Given that female reproductive dysfunction and related disorders are classic features of obesity and insulin resistance [61], our data suggest that pharmacological incretin receptor modulation could represent a novel non-invasive means for treating energy related reproductive disorders.

## Figures and Tables

**Table 1:** List of primers.

Gene Symbol	Alias/Common Name	Primer Sequence (5′-nt-3′)	Product Size	Annealing Temperature
**GIPR**	*Gastric inhibitory polypeptide receptor*	Forward: CTACTCCCTGTCCCTGACGA Reverse: AGCTGATCTCGGGTGAGGAT	147	57
**GLP-1R**	Glucagon-like peptide 1 receptor	Forward: TCACTTCCTTCCAGGGCTTG Reverse: CACTTGAGGGGCTTCATGCT	145	57
**ACTB**	Actin, beta	Forward: GAGCGCAAGTACTCTGTGTG Reverse: AACGCAGCTCAGTAACAGTC	152	57

## Data Availability

Data is contained within the article.
